# Hybridity in the housing sector: examining impacts on social and private rented sector tenants in Scotland

**DOI:** 10.1080/02673037.2019.1648770

**Published:** 2019-08-07

**Authors:** Steve Rolfe, Lisa Garnham, Isobel Anderson, Pete Seaman, Jon Godwin, Cam Donaldson

**Affiliations:** a Faculty of Social Sciences, University of Stirling, Stirling, UK;; b Glasgow Centre for Population Health, Glasgow, UK;; c School of Health and Life Sciences, Glasgow Caledonian University, Glasgow, UK;; d Yunus Centre for Social Business and Health, Glasgow Caledonian University, Glasgow, UK

**Keywords:** Housing, homelessness, hybridity, social housing, private rented sector, social enterprise

## Abstract

Housing Associations in many countries exhibit increasing levels of ‘hybridity’, as reductions in state financing for social housing, exacerbated by austerity policies since the 2008 crash, have instigated ‘enterprising’ approaches to maintaining income. Alongside this, hybrid organisations have emerged in the Private Rented Sector (PRS), responding to sectoral growth and consequent increases in vulnerable households entering private renting. These developing hybridities have been considered at a strategic level, but there has been little exploration of the impacts on tenants. This article examines two organisations, operating across the social and private rented sectors, to elucidate potential implications for tenants. The research suggests that different forms of hybridity can affect tenant outcomes and, moreover, that examining such impacts is important in understanding hybridity itself. Furthermore, the study suggests that emerging forms of hybridity, particularly in the PRS, may be blurring the boundaries between housing sectors, with implications for policy and research.

## Introduction

The notion of hybridity has been widely applied to examine developments in organisational form, structure and strategy, particularly in relation to the involvement of third sector organisations and private companies within the ‘welfare mix’ (Billis, 2010; Buckingham, 2011). Whilst there is considerable debate about the existence of non-hybrid ‘ideal types’, to the extent that some authors contend that hybridity has become the norm (Brandsen *et al*., 2005; Evers, 2005), the concept provides a useful lens through which to explore the ways in which internal and external drivers may shift organisations towards or away from the distinctive characteristics of market, state or community sectors.

Within the housing literature, hybridity has primarily been utilised to explore changes in social housing organisations. In particular, Housing Associations have been examined as organisations which are both inherently hybrid (Blessing, 2012) and subject to particular regulatory and financial pressures which alter their manifestations of hybridity (Czischke *et al*., 2012; Morrison, 2016; Mullins *et al*., 2012, 2017). However, the attention paid thus far to hybridity in housing organisations focuses almost exclusively on strategy and structure, examining the organisational impacts of market and state drivers (Gruis, 2008; Mullins, 2006; Mullins & Jones, 2015). Whilst there has been some recognition that hybridity involves the development of hybrid housing products, such as shared ownership and renting at market or near-market prices (Gilmour & Milligan, 2012; Gruis, 2008; Morrison, 2016), there is a substantial gap in the literature in terms of impacts of hybridity on tenants. Furthermore, since hybridity has been largely applied in studies of social housing organisations, the relevance of the concept to housing providers operating in the Private Rented Sector (PRS) has not been examined.

This article attempts to address this gap by specifically examining the impacts on tenants of different forms of hybridity within two housing organisations, operating in the social and private rented sectors. The organisations are based in Scotland, which provides an interesting context for studying hybridity because of pressures from regulatory change and sectoral shifts which are arguably leading to convergence between social housing and the PRS. The particular lessons from this context are likely to be of value more broadly, given common experiences of austerity, marketisation and therefore hybridisation internationally (Poggio & Whitehead, 2017).

The next section provides a more detailed discussion of hybridity within the existing housing literature, and outlines the background to the study in terms of Scottish housing policy and sectoral balance. The subsequent section outlines the methods employed in the research. The case study organisations are then described and their hybrid characteristics explored, using data from staff interviews. This is followed by an exploration of the data from tenants, focusing particularly on impacts of different elements of hybridity. The article concludes by discussing these findings in relation to the wider literature, with some thoughts about the implications for housing policy and research.

## Context

### Defining hybridity as an analytical frame

The notion of hybridity is still somewhat emergent and elusive (Mullins *et al*., 2012), with multiple subtly different definitions. Indeed, the more critical view of hybridity presents it as ‘a concept that is widely used but seems to play no useful function in theory building or advice to policy-makers’ (Skelcher, 2012). The difficulties here are essentially twofold. First, there are differing perspectives with regard to the number and definition of ‘non-hybrid’ sectors between which aspects of hybridity emerge. Some authors conceive of hybridity along a linear spectrum between the two poles of state and market organisations, or social and economic drivers (Blessing, 2012; Crossan & Til, 2009), whilst others present a triangular model, with ‘community’ or ‘civil society’ providing the third corner to complement state and market (Billis, 2010; Evers, 2005). The latter models add further complexity, since some present ideal-typical third sector organisations (TSOs) as existing at the non-hybrid community vertex (Billis, 2010), whilst others conceive of all TSOs as being in a ‘tension field’ between state, private and community sectors (Evers & Laville, 2004).

Second, there is considerable diversity in approaches to characterising hybridity. Whilst there is some commonality in considering hybridity as a phenomenon of mixing or departing from the acme of state, market and sometimes community, there is far less agreement on the nature of ideal-type organisations for each sector, or their analytical usefulness when most organisations display elements of hybridity (Brandsen *et al*., 2005; Buckingham, 2011). Moreover, hybridity is understood as a dynamic process in reaction to different pressures or drivers (Billis, 2010; Evers, 2005), making it difficult to characterise or categorise particular forms of hybridity within organisations (Crossan & Til, 2009).

Hence, discussions of hybridity risk relying on ill-defined concepts, or demonstrating little more than the extreme rarity of non-hybrid organisations (Skelcher, 2012). Despite these challenges, however, the notion of hybridity offers considerable value in housing research, partly because of the distinctive nature of housing itself. As Blessing (2012) has suggested (drawing on Bengtsson (1995)), the status of housing as both market commodity and public good requiring state involvement creates a focus on state/market tensions. Moreover, processes such as reductions in state funding/subsidy for social housing and transfer of public housing stock to housing associations act as drivers of hybridisation (Blessing, 2012), albeit that state control may continue and value-based TSO identities may resist marketisation (Buckingham, 2012; Mullins *et al*., 2017; Nieboer & Gruis, 2014).

Thus, hybridity has been usefully employed to examine the growth and evolution of housing associations, highlighting the ways in which third sector housing providers face conflicting priorities arising from their charitable values, market pressures and state regulation (Gruis, 2005; Morrison, 2016; Mullins *et al*., 2012). Alongside this, hybridity also offers a conceptual frame to examine diverse policy drivers incentivising entrepreneurial, market-focused approaches in housing organisations in high-income nations, including the US, Australia and across Europe (Bratt, 2012; Czischke *et al*., 2012; Gilmour & Milligan, 2012), whilst demonstrating different forms of state-market interaction in countries such as South Korea and China (Lee & Ronald, 2012; Wang & Murie, 2011). To examine organisational responses across such diverse contexts, ideal types need to be seen not as empirical reality, but as analytical tools to explore hybridisation processes (Skelcher, 2012). Hence, as Billis (2010) argues, the value of comparing characteristics such as ownership, governance, operational priorities, and human and other resources with ideal types lies in identifying how particular organisations are moving into different “zones of hybridity”, combining principles derived from public, private and third sectors.

Notably, this literature focuses largely on impacts at the level of organisational strategy, structure and governance, rather than potential effects of hybridity on frontline services and, ultimately, on tenants. Moreover, while the more recent emergence of ‘enacted’ hybrid organisations (Billis, 2010) in the form of socially-focused letting agencies operating in the PRS has been descriptively explored in national contexts across Europe (De Decker, 2002, 2012; Hegedus *et al*., 2014; Laylor, 2014; Mullins & Sacranie, 2017; Shelter Scotland, 2015), there has been little examination of their hybridity, or the implications for frontline services and tenants.

Importantly, this literature also points to an ambiguity in the conception of hybridity as applied to housing organisations. As Lee & Ronald (2012) suggest, it may be useful to consider not only aspects of ‘organisational’ hybridity, relating to aspects such as resources, governance or legal form, but also ‘modal’ hybridity, examining the extent to which housing products blend aspects of social/public housing, or market/PRS models, in areas such as rent level, allocation or tenure (Morrison, 2016). Clearly there are strong connections between these two aspects of hybridity, since housing providers which exhibit organisational characteristics closer to the public sector, for example, are more likely to deliver housing products which approximate the ideal type of social housing. However, the dynamic and complex nature of hybridity within organisations precludes any simple correspondence between organisational and modal hybridity. Moreover, the distinction is particularly important in terms of potential impacts on tenants, since it seems reasonable to hypothesise that tenants will be more concerned with, and directly affected by, the housing product rather than the nature of the organisation.

The definition of social housing and therefore the distinction between social and private rented housing is debated, since official definitions vary between states and evolve over time (Granath Hansson & Lundgren, 2018; Oxley, 2000; Scanlon *et al*., 2014). However, there is considerable commonality across the literature in examining issues of allocation, rent levels, subsidy, ownership and regulation as useful to categorise housing as social or private rented. Hence, in researching hybridity within housing organisations, there is value in examining organisational aspects of hybridity, such as those suggested by Billis (2010) and these elements of modal hybridity in terms of housing products. This article takes such an approach, describing the participant organisations in these terms before considering their implications for tenants.

### The Scottish context

**Figure 1.**
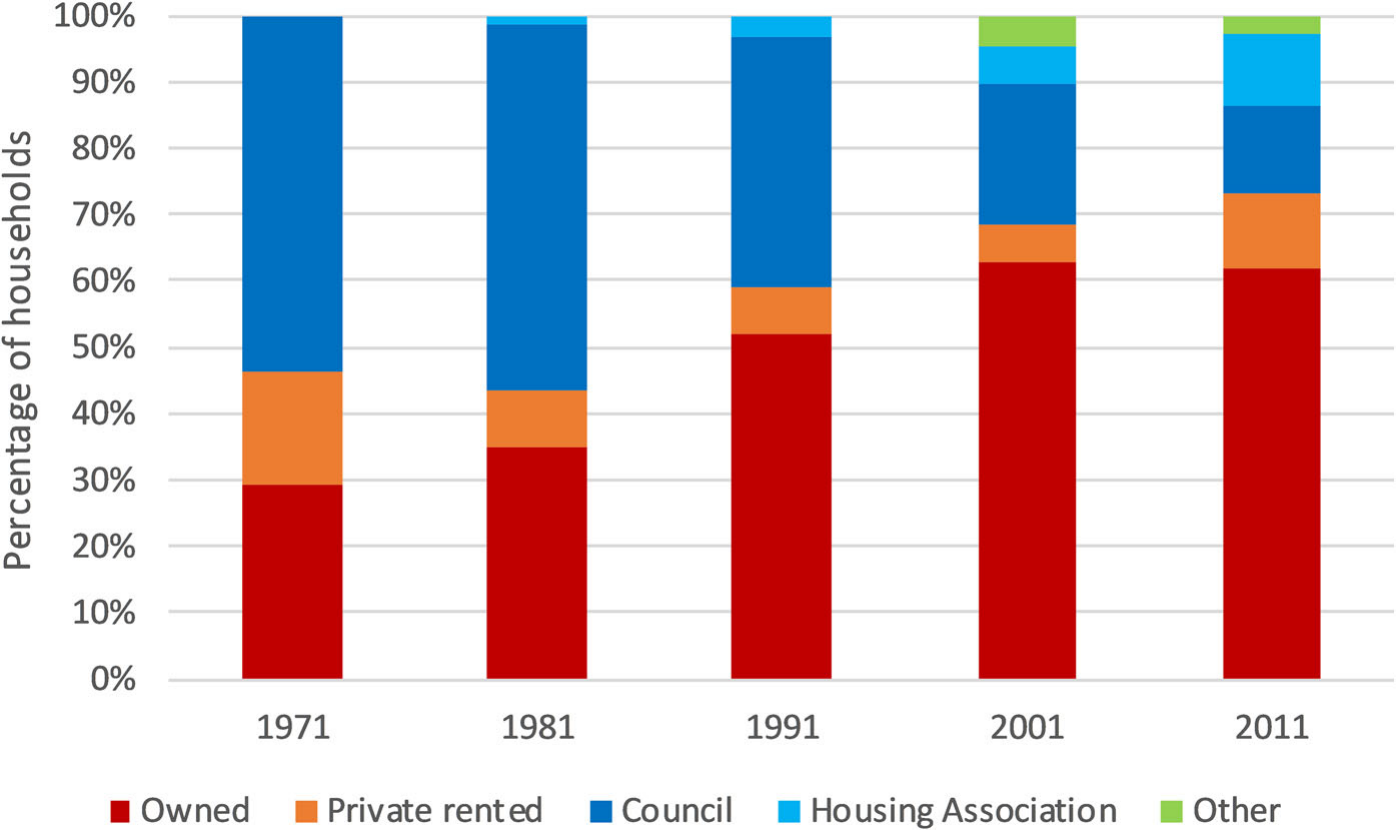
Housing sectors in Scotland, 1971–2011. (Source: Census data).

Changes in the sectoral balance and regulation of the Scottish housing system are relevant in considering the development of hybridity. As Figure 1 shows, the last half-century has witnessed a shrinking social housing sector as a result of the Right to Buy policy^1^, as well as a shift from state provision to the third sector, following stock transfers^2^ from some local authorities, most notably Glasgow. Alongside this, owner occupation growth has largely stalled since 2000, whilst the PRS has more than doubled in size, now accounting for around one in six households.

Thus, whilst the most vulnerable households, particularly those leaving situations of homelessness, tend to be housed primarily in the social housing sector, the limited stock is unable to meet demand. As a result, there is increasing evidence of growing numbers of low-income and vulnerable households in the PRS (Bailey, 2018).

Alongside this, there are notable changes in PRS regulation in Scotland, arising partly as a response to these sectoral shifts. The Scottish Government has introduced the Private Residential Tenancy, making all new PRS tenancies open-ended and removing ‘no-fault evictions’, as well as schemes of registration and regulation for PRS landlords and letting agents, changes to dispute resolution mechanisms for tenants and protection for tenancy deposits^3^. Whilst regulation systems remain distinct for the PRS and social housing sectors, these changes bring tenant security in the PRS somewhat closer to that in social housing.

This study attempts to examine the impacts of hybridity for tenants of housing organisations within this context, thereby elucidating some of the potential outcomes arising from these apparent convergences between social housing and the PRS in Scotland.

## Methodology

The data for this article is taken from a longitudinal, mixed methods study of the health and wellbeing impacts of different approaches to housing provision in three organisations, although this article draws on data from just two^4^. The study consisted of three phases.

In the first phase, interviews were carried out with 13 staff from the organisations, to clarify their approach to housing provision and relevant aspects of hybridity. The second phase involved three waves of data collection from a cohort of new tenants within each organisation, over the period 2016–2018. Data was collected through structured interviews carried out at the start of the tenancy (wave 1), focused on background data regarding previous housing experiences, then at 2–4 months (wave 2) and 9–12 months into the tenancy (wave 3). Quantitative data was collected at all three waves, whilst qualitative data was collected at waves 2 and 3, examining four aspects of tenants’ housing experience (housing service, property quality, affordability, and community and social networks) as well as health and wellbeing, financial circumstances and demographics. Table 1 sets out the numbers participating at each wave^5^. The final phase of the study involved focus group discussions with staff, to further examine the approach of each organisation in the context of the data from tenants.

**Table 1.** Numbers of participating households at each wave.

Organisation	Wave 1	Wave 2	Wave 3
Housing association	56	33	23
Letting agency	50	34	17
Total	106	67	40

The data from staff interviews and focus groups was analysed using Nvivo, employing a coding framework derived from characterisations of hybridity in the literature. The next section of the article provides a detailed introduction to the organisations and outlines the findings from this analysis. The subsequent section utilises descriptive statistics from the quantitative data (analysed using SPSS) to set out the key outcome patterns for tenants of each organisation, and then uses the qualitative data from waves 2 and 3 (analysed using Nvivo) to explore the potential links between aspects of hybridity and tenant outcomes. This article does not focus on the longitudinal aspect of the study beyond highlighting the impacts in terms of health and wellbeing, so quotes are identified by organisation, but not by wave.

## Hybridity within the participant organisations

### Staff perspectives

This section introduces the participant organisations and outlines their key characteristics in relation to aspects of organisational and modal hybridity, drawing on the staff interviews and focus groups, together with documentation where appropriate.

The first organisation is a large Community-Based Housing Association, formed by tenants in the mid-1970s in response to demolition plans for their Council houses. It now has over 5000 properties, around half of which were acquired through stock transfer within the last decade. The organisation operates as a relatively traditional social housing manager, not engaging with innovations supported by the Scottish Government’s Affordable Housing Supply Programme (Scottish Government, 2016), such as Mid-Market Rent^6^.

The second organisation is a social enterprise Letting Agency, set up in 2013 by its current Director, consisting of two connected companies. The Letting Agency wing manages property for PRS landlords, but is not-for-profit, unlike most letting agencies. The Investment wing purchases properties using social investment loans, renovates them and rents them through the Letting Agency. Both wings operate with a social mission to provide high quality housing within the PRS to vulnerable and low-income households. The Investment wing owns just over 200 properties, whilst the Letting Agency manages another 250 for private landlords.

Table 2 summarises the structure and operation of the two organisations, utilising a combination of Billis’s (2010) five core elements, and attributes used to differentiate social and private rented housing provision (Granath Hansson & Lundgren, 2018; Scanlon *et al*., 2014). This characterisation of the organisations therefore attempts to include behavioural attributes (e.g. housing products) alongside structural descriptors (e.g. governance, ownership) and motivators (i.e. operational priorities). As Crossan & Til (2009) have argued, behavioural indicators are essential in classifying organisations in terms of hybridity. Because ownership and financial resources/subsidy appear in both lists, this gives eight characteristics in total.

**Table 2.** Characteristics of participant organisations.

	Housing association	Letting agency
Ownership	Community Benefit Society, formally owned by members, who elect a Board. Membership of the organisation is open to anyone within the local community, not just tenants. Board members primarily drawn from membership, with a small number of co-opted places to fill skills gaps.	Letting Agency is a Community Interest Company, owned by shareholders. Articles preclude profit distribution to shareholders and restrict asset disposal. Investment wing is a Company Limited by Guarantee, owned by Letting Agency (40%), Director (40%) and Social Investment Firm (20%).
Governance	Day-to-day decisions taken by Executive Team of staff. Oversight by Board, with input from wider group of tenants via Area Committees.	Day-to-day decisions taken by staff team, managed by Director. Oversight by Board, which includes Director.
Operational priorities	Mission is ‘To provide quality homes and on-going community regeneration and empowerment’.	Mission is ‘To provide quality lettings with the aim of establishing sustainable, affordable, long-term housing options for all tenants, in particular those in housing need, those on low incomes or in receipt of benefits’.
Human resources	Staff team of more than 120 full-time equivalent posts, managed by Executive Team.	Staff team of around 10 people across the two wings.
Other resources	Income derived primarily from rent. Historic subsidy from state in the form of Housing Association Grant. More recent funding in loan form, primarily from private sector lenders.	Income derived primarily from rent. Loan funding for property purchase from social investment company. Grant funding to support employment of tenancy support staff.
Allocation	Properties allocated using points-based system, giving priority to households in greatest need. Direct referrals of homeless households from the local authority fill around 15% of vacant properties each year.	Properties advertised publicly – prospective tenants apply for a particular property. Properties owned by investment wing allocated on the basis of tenant need, although with financial assessment. Private landlord properties allocated on the basis of ability to pay, although with some assessment of tenant need, depending on individual landlord.
Rent levels	Rents set below market levels. Long-standing tenants have significantly lower rents, whereas rent for new tenancies is much closer to market levels. All rents subject to same annual percentage rise, so harmonisation only occurring through change of tenancies.	Rent levels for Investment wing properties capped at no more than 5% above Local Housing Allowance rates. Rent for private landlord properties set by market/landlords.
Regulation	Regulated by the Scottish Housing Regulator as a Registered Social Landlord. Required to meet the standards in the Scottish Social Housing Charter, which covers customer relationships, housing quality, neighbourhood management, value for money, and access to housing and support, and to ensure that properties meet the Scottish Housing Quality Standard.	Letting Agency subject to registration and required to meet statutory Code of Practice. All properties required to meet PRS Repairing Standard. Landlords subject to registration and required to meet fit and proper person test.

It is clear that the Housing Association exemplifies a relatively traditional social housing manager (Gruis, 2008), primarily focused on meeting the housing needs of its social disadvantaged tenant group. Nevertheless, the shift from grant to loan finance demonstrates a degree of hybrid financial dependency (Morrison, 2016), whilst the large-scale stock transfer of housing from public ownership, along with a number of public sector staff, can also be viewed as a process of hybridisation, developing aspects of managerial, state-bureaucratic structure and behaviour (Blessing, 2012). Setting aside the question of whether Housing Associations, as TSOs, are inherently hybrid, these elements of emerging hybridity through policy change suggest that the Housing Association exhibits ‘organic’ hybridity (Billis, 2010) as the organisation has grown and developed over time.

The Letting Agency provides a more explicit example of hybridity, melding non-profit and social mission characteristics into a type of enterprise which is usually profit-driven, and exhibiting modal hybridity in the form of rent restrictions and priority for low-income households in allocating owned properties. As Brandsen *et al*. (2005) suggest, such organisations ‘on the fringe’ are empirically valuable in understanding processes, forms and impacts of hybridity. Moreover, the Letting Agency exemplifies ‘enacted’ hybridity (Billis, 2010), having been created by its Director in its current form.

Evidence from staff interviews and focus groups indicate how each organisation is influenced by private, public and third sector principles, shaping particular manifestations of organisational and modal hybridity.

As a social landlord with origins in community activism, the Housing Association’s priorities are shaped by third sector principles, focusing on affordability and housing needs of vulnerable and low-income households:

Dedication to offering housing solutions and routes into social inclusion by building, managing and maintaining a range of affordable housing, and providing support for varying needs (Housing Association, Strategic Aims).

However, whilst it sets rents below market rates, the influence of market pressures is evident in the higher rents for new tenants than for long-standing tenants in equivalent properties. Moreover, all rents are increased by a set percentage, decided on by the Board following a tenant consultation, which therefore does not reduce the differential and has a greater absolute impact on newer tenants. These higher rents create concerns about affordability and tenancy sustainability in a context of welfare reform:

There are varying reasons [why people move on] as you can imagine…affordability can be a reason as well. And not necessarily meaning that our rents are unaffordable. I think it’s more about some people move back to family because of all the cuts and changes in benefits. (Housing Association, Assistant Director of Housing Services)

These financial drivers also combine with bureaucratic structures and values which have developed through organisational growth and the transfer of public sector staff. Hence, for example, property refurbishment prior to a new tenant moving in is restricted by bureaucratic commissioning systems and the risk of financial loss if tenancies are not sustained:

If it’s somebody that’s older, we’ll maybe see if we can paint a room or do something. But the costs are astronomical for us to be able to paint a room… Because when you’re paying contractor rates, you know. So the difficulty is, people think that, I could get that done for £100, so add it onto my rent. And how long do they stay, you might say, well add it over the course of 2 years, and they stay 2 months… so it's quite difficult. (Housing Association Manager)

Ultimately, this leads to a managerial focus on the housing stock as a higher priority than the immediate needs of tenants:

And the sad thing is, from a housing perspective, we’re really concerned, obviously, we’re concerned about the tenants, of course, and that’s a given. But it's our house, it's our income, and that’s the thing that we should be concerned about. (Housing Association Manager)

There is evident tension, therefore, between the community-focused third sector principles embodied in the Housing Association’s mission statement, private sector principles arising from the removal of public subsidy, and public sector principles emerging from regulation and stock transfer.

For the Letting Agency, third sector principles also underly the organisation’s social mission, summarised by the Agency’s founder as:

to ensure that… vulnerable people get access to quality housing and are treated well. (Letting Agency, Director)

However, although this social mission applies across the organisation, the deliberately hybrid nature of the organisation leads to some differences in priorities and operation between the two wings, indicating tensions with private sector principles in particular.

In terms of rent levels, for the properties owned by the Agency rents are capped at no more than 5% above the Local Housing Allowance rate^7^, whilst rents in the private landlord properties are set at market rates. Thus, third sector principles keep rents on Agency-owned properties within a nominally ‘affordable’ range, but there is a recognition that these are still somewhat higher than for equivalent social housing:

it can be quite tricky if people are waiting to get a Housing Association property because obviously the price of [our] rent is higher, so that can freak people out a bit (Letting Agency, Assistant Director)

Moreover, whilst the organisation aims to work with sympathetic landlords, there is a particular tension with private sector principles in the need to retain business by ensuring that landlords profit financially:

really the main aim is to create happy homes and sound investments for landlords, so obviously we want the landlords to know that they’re getting the best possible quality service for the price that they pay, and that we’re doing what we should be to ensure that… tenants are fit and proper to be going into the property (Letting Agency, Assistant Director)

This is particularly important because the private landlord side of the business is intended to provide a degree of cross-subsidy for tenancy support, which primarily assists vulnerable tenants in Agency-owned properties.

This tension also arises in relation to property condition, where the Agency employs an interior designer to deliver high quality refurbishments in its own properties, but has to balance its mission to provide quality housing with the need to grow its private landlord business:

We try to provide homes at the highest standard we can. The ones that we own, we have direct control over the quality of the décor and the finishing and the safety and all of that. When it is landlords that we are working with we have less control and there have been landlords that we have turned away because the quality wasn’t acceptable. (Letting Agency, Director)

For the Letting Agency, therefore, there is clear evidence of tension between third sector and private sector principles. Unlike the Housing Association, however, this is less about hybridity emerging over time, but rather an inherent tension, with the more market-focused aspects being designed to financially underpin the socially focused mission.

### Tenant demographics

The evidence regarding tenant demographics also provides some indication of drivers for hybridity, in terms of their manifestation in allocation processes and outcomes. Table 3 provides demographic characteristics for the tenants of each organisation^8^. The data for the Letting Agency is split between tenants in properties owned by the organisation and tenants in private landlord properties, given the differences in approach outlined above.

**Table 3.** Demographic characteristics of tenants.

Characteristic		Housing association	Letting agency	
			Owned	Private landlord
Age	Younger (<35)	36%	38%	78%
	Older (≥35)	64%	63%	22%
Disability	Disabled	42%	25%	6%
	Non-disabled	58%	75%	94%
Employment	Employed	24%	69%	67%
	Not employed	76%	31%	33%
Household type	Household without children	64%	69%	83%
	Household with children	36%	31%	17%
Household income (AHC)	<50% median	91%	75%	50%
	50–60% median	3%	6%	17%
	60–100% median	6%	19%	22%
	>100% median	0%	0%	11%
Housing Benefit	Full or partial Housing Benefit	76%	38%	6%
	No Housing Benefit	24%	63%	94%
Previous housing situation	Social housing	27%	13%	0%
	Private rented sector	24%	56%	67%
	Homeless	30%	6%	6%
	Other	18%	25%	28%

The higher levels of disadvantage amongst Housing Association tenants compared to Letting Agency tenants in private landlord properties, in terms of the proportion who are disabled, out of work, or on a low income, suggests an impact of prioritisation through allocation systems, as would be expected between social housing and the PRS. The much higher proportion of Housing Association tenants coming from homelessness reflects the role of “Section 5 referrals”, whereby the local authority can refer homeless households to Housing Associations^9^. Meanwhile, the intermediate levels of disadvantage for tenants in Letting Agency-owned properties indicate the effect of priority being given to vulnerable households for these properties, underpinned by a condition of the social investment loans requiring 75% to be rented to vulnerable or low-income households. Clearly there may be other factors at play here, such as the characteristics of the Housing Association’s area, which is entirely within the most deprived quintile of the Scottish Index of Multiple Deprivation (Scottish Government, 2017). Nevertheless, these tenant demographics suggest a significant influence of third sector principles in the allocation processes of both organisations, alongside effects of public sector regulation for the Housing Association and market pressures for the Letting Agency.

This evidence from staff interviews and tenant demographics provides an initial indication of processes of hybridisation operating within each organisation, demonstrating the value of studying these two organisations to examine hybridity and its potential impact on tenants. Exploring tenant outcomes within these organisations may elucidate impacts of different aspects of organisational and modal hybridity across social and private rented sectors. The aim within this article is to examine what can be learned about impacts of hybridity within each organisation as specific examples of organic and enacted hybridisation, using the comparison between the organisations and between the tenant groups within the Letting Agency to delineate these impacts.

## Impacts of hybridity – tenant experiences and outcomes

Perhaps unsurprisingly, little evidence emerged that tenants were significantly affected by aspects of ownership and governance, despite the priority given to these in studies of hybridity. Indeed, most tenants seemed largely unaware of these aspects of their housing organisation. However, other aspects of the tenant experience, shaped by the ways in which the tensions described above play out in practice, demonstrate explicit and implicit links to most of the other aspects of hybridity. This section considers the impacts of hybridity on key aspects of tenants’ housing experience: tenancy affordability; property quality; and housing service and tenure.

### Affordability

**Figure 2.**
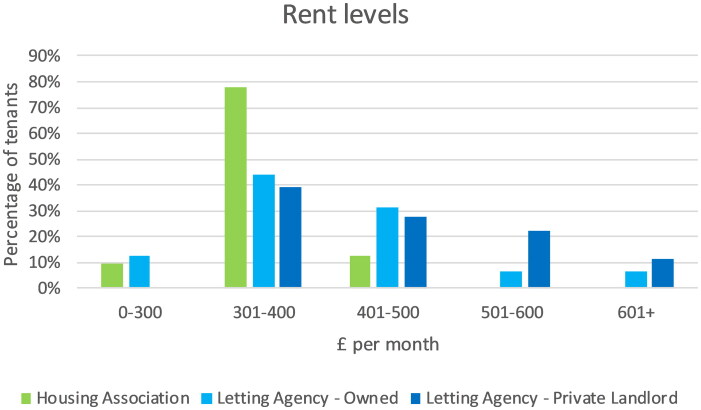
Rent levels.

Financial drivers are clearly important alongside third sector principles in terms of rent levels, but these feed through into organisational practices and impacts on tenants in different ways. Figure 2 summarises rent levels of participating tenants, demonstrating the generally lower rents for Housing Association tenants, with private landlord properties showing somewhat higher rents within the range of Letting Agency properties.

In terms of outcomes, however, rent levels clearly interact with the financial circumstances of tenants. More than 90% of tenants across both organisations described themselves as coping with rent payments all or most of the time, although for somewhat different reasons. Nearly 60% of Housing Association tenants had their rent entirely covered by Housing Benefit, whereas only 6% of Letting Agency tenants received full Housing Benefit, but most were able to cover their rent from their higher incomes.

Where tenants do fall into arrears, however, the qualitative data suggests that the organisations operate different approaches. For the Housing Association, financial pressures, regulatory requirements regarding financial management and the scale of the organisation create an approach which is experienced by tenants as being relatively inflexible:

They are filing a court case against me because I was unable to pay my rent, sincerely speaking I didn’t pay in July… I made a payment in September… but according to them that’s not their protocols (Housing Association tenant)

By comparison, the Letting Agency is able to operate a more flexible approach in relation to its own properties, reflecting a stronger financial position and freedom from regulation around financial management and risk:

[Letting Agency staff member] says, so long as you can make your shortfall, it doesn’t matter that you’re paying a couple of pounds a month or whatever towards your arrears, that £800. I mean… you can increase it over the next 2/3/4 years. So even with him saying that – ‘2/3/4 years’ – then straightaway it, kind of, grounds me a wee bit more. I’m not… getting turfed out on my ear and things like that, so peace of mind and security. (Letting Agency tenant)

Rent levels also interact with tenants’ expectations, with Letting Agency tenants generally accepting their rent as the market rate. Whilst Housing Association tenants were largely coping with their rent, a small minority did raise concerns about the rent level. For some, this was about rent differentials within the organisation:

I pay a lot more than what she does up the stair cause apparently their rents were frozen, she's been there that long… and her rent’s frozen at £270 something. (Housing Association tenant)

Perhaps more notably, for a few tenants, the difference between Housing Association and market rent levels in the area was small enough that they would consider moving to the PRS to overcome other concerns about their tenancy:

I probably want to go with private renting. Everybody always says to me that it was daft to go private, the council’s much better, the housing associations are much better, my experiences haven’t been, so I don’t think a private landlord can be any worse, to be fair… Housing Associations used to be much cheaper, now they’re not. I mean, I can get… a two bedroom for 450, so I’m going to be paying 50 pounds more a month. (Housing Association tenant)

Hence, whilst Housing Association rents remain below market levels, this suggests that the financial pressures on the organisation have enforced a degree of adherence to private sector principles, raising rent. to a level almost comparable to PRS rents, from a tenant perspective.

### Property quality

**Figure 3.**
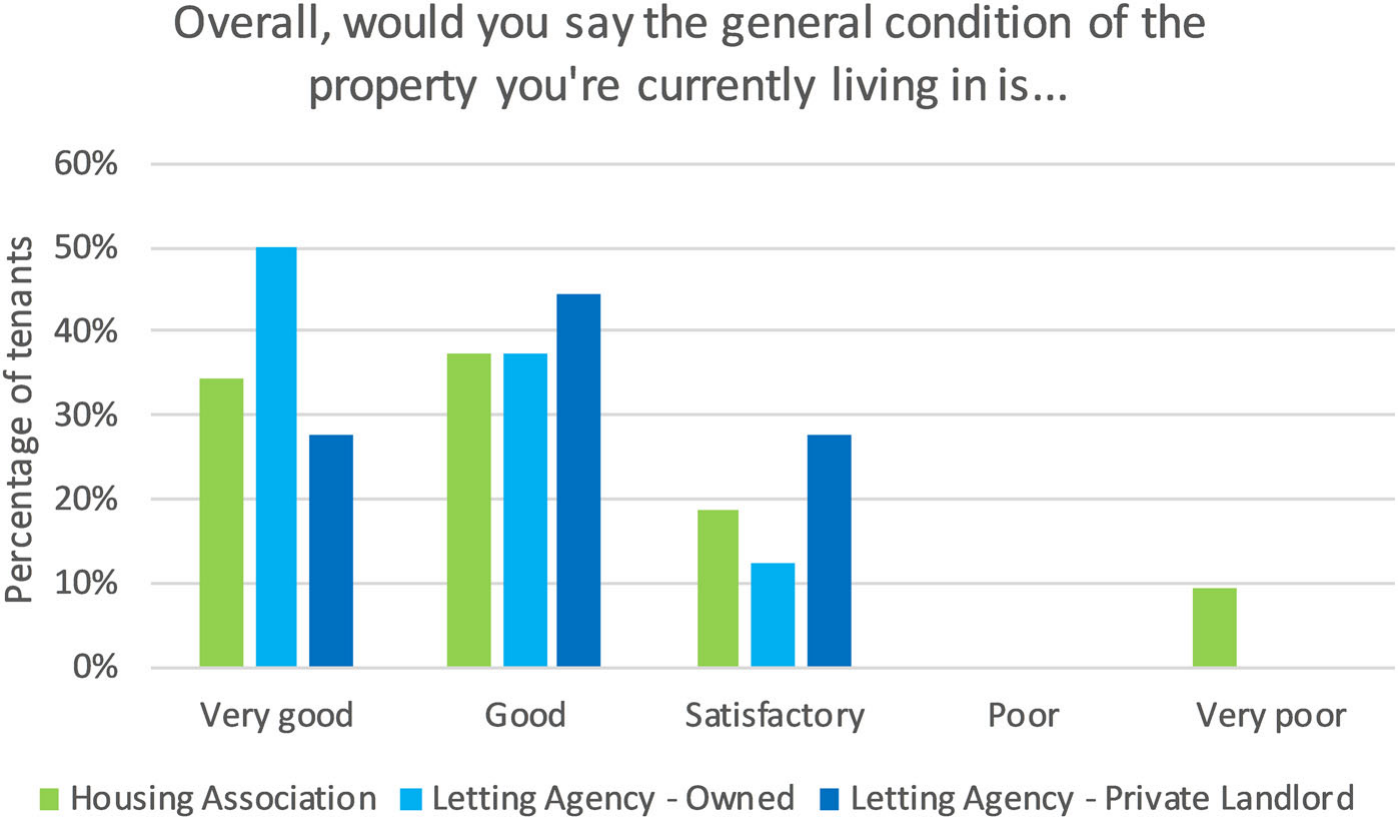
Tenant rating of property quality.

Tenant perceptions of property quality at the move-in point are quite similar across the organisations, as shown in Figure 3. The main differences that emerge are the higher proportion of tenants in Letting Agency-owned properties rating them as “very good” and the small number of Housing Association tenants rating their property as “very poor”. The figures are not directly comparable across the sectors, since Housing Association properties are let unfurnished, whereas Letting Agency properties are generally furnished. However, the evidence from tenant’s points towards notable differences in organisational approach and underlying drivers.

Whilst the majority of Housing Association tenants were relatively happy with their property, the move-in condition depends largely on how the property was left by the outgoing tenant, and expectations are clearly important in terms of tenant's perspectives. In some cases, this was positive:

I had a feeling that it could have been worse. But when they opened it, I thought this was a show home… I've seen, I've been in houses that…this is at the top. I thought it would have been worse. I had all different things going on in my head, until she opened the door. And I went, oh wow. (Housing Association tenant)

Staff recognised the potential value of greater investment in refurbishment for new tenants where the previous tenant had left the property in a relatively poor condition, but significant financial and bureaucratic constraints largely preclude such work, as outlined earlier. Hence, some new tenants were very disappointed and, in the worst situations, this risked undermining their tenancy altogether:

Like, the walls in here are pretty bad and at one point I phoned the housing officer and I says to her, listen, I'm going to have to give you that house back. That’s far too much work for me… I’ve nobody to help me or nothing and…there’s nothing I can do to that house. And I ended up saying to her, I'm going to end up just giving you your keys back, ‘cause I can’t cope. (Housing Association tenant)

For tenants in Letting Agency-owned properties, there was a clear impact of third sector principles prioritising the quality of refurbishment, particularly where this contrasted with previous experiences. Thus, property quality helped tenants to settle in and avoid additional expenditure:

Aye, top notch standard… basically everything in here apart from this, that and that was all here – couch, table, chair, fridge, everything you see was all here, very, very nicely furnished when I moved in so I didn’t have to do anything to it, just move my stuff in and find a space for it, that's it. (Letting Agency tenant)

I like the fact that the flat was walk-in condition and… I didn’t have that expense of putting new floors, new carpets, and all that, and because I wouldn’t take the kids into a place where someone…because you don’t know whose been in it before, so I’m a bit freaky about that… That was a big expense that I didn’t have that allowed me to…I can save up now, I’m starting to be able to save money rather than having to get it decorated. (Letting Agency tenant)

The regulatory minimum standard for property quality is higher for social housing providers (the Scottish Housing Quality Standard) than for PRS landlords (the Repairing Standard). In this instance, however, financial and bureaucratic pressures prevent the Housing Association lifting properties above the minimum, whereas the Letting Agency utilises its financial flexibility to invest in consistently high-quality refurbishment.

### Service and tenure

**Figure 4.**
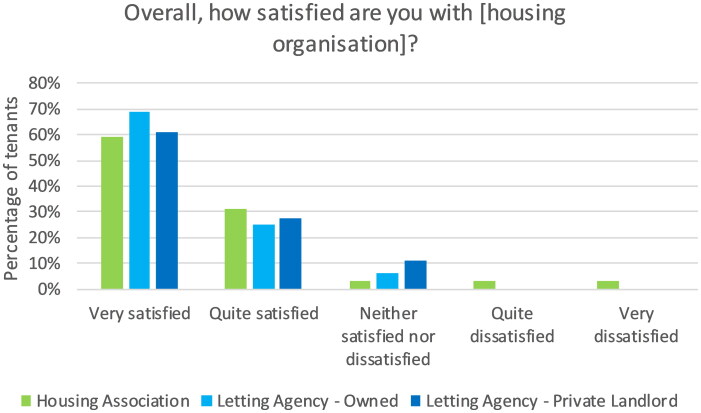
Tenant satisfaction with organisation.

Both organisations emphasise the importance of providing high quality customer service and support to tenants, evidenced through the Customer Service Excellence Award held by the Housing Association and the Letting Agency’s investment in its Tenancy Support service. It is unsurprising, therefore, that tenants across the organisations give them high satisfaction ratings, as shown in Figure 4.

The qualitative data inevitably shows a more nuanced picture, with previous experiences and expectations playing a key role in shaping tenants’ perspectives across both organisations:

I started my career with social housing… so I do know a bit about social housing, but [this Housing Association] have been really, really good… Hundred times better than I thought it was going to be. (Housing Association tenant)

[They’re] obviously really good at what they do, they’re not overbearing, some letting agencies can be and they can be quite rude as well, whereas [this Letting Agency] have always been 100% honest, genuine, nice people. I mean, they want to see you do well and be comfortable in a house that they’ve rented you, so their attitude just seems a lot friendlier and they show a lot more concern for their residents than anybody else that I’ve come across. (Letting Agency tenant)

For those tenants of the Housing Association who were dissatisfied, the central factor seemed to be communication, related to the scale of the organisation:

And I ended up having to deal with [the repair issue] when I was in my work, and I was crying down the phone. I was like, I'm so stressed out at repeating myself; and different people telling you different stories all the time… So, at the start of this I was dealing with one housing officer, but then she left and the new one was yet to be here. So, I don't know if that's maybe made a difference? There's not one person dealing with it. (Housing Association tenant)

Across both organisations, satisfaction was at least partly related to tenants’ sense of security in their tenancy. For Housing Association tenants, this was underpinned by the security of a lifetime tenancy agreement:

Once you go over the door it’s just like, do you know this is my flat, it feels good…. because when we were looking at private lets, it was like renewing contracts and stuff like that which was kind of daunting. Whereas as long as we make our end of the deal then the flat's ours. (Housing Association tenant)

For tenants in properties owned by the Letting Agency, the reassurance given by staff created a similar impact, particularly with positive communication at the end of the initial, standard 6-month tenancy period:

I think I feel better in general, I suffer from anxiety and stress in the past, I was also on medication, that was before last year, it was 2 years ago, and I would definitely say that that has improved… [the housing is] definitely one of the factors, you know, not having to stress about where you live is a good thing. (Letting Agency tenant)

Indeed, for some tenants, the Letting Agency seemed more like a social housing provider:

In previous houses, private lets and that and… I didn’t have the same service, kind of thing, you know. It’s just a totally different group that I’m working with this time, the housing association… I haven’t heard of a housing association like them where they’ll actually come out and, you know, be as hands on with their tenants and…in a positive way rather than pressuring the tenants. (Letting Agency tenant)

For tenants across both organisations, therefore, the priority given to customer service and tenancy support provided a positive, secure housing experience which helped to underpin a sense of home. This in turn led to improvements in tenants’ overall quality of life and, ultimately, their health and wellbeing:

Because I’m comfortable in here, eh, I can go and start doing things, like some acting, you know, or even just go for a walk, or a drive, or jump on a bus. You know, 'cause I'm not in a lot. Because I am still pretty new to Glasgow, so, 'cause I still have the free bus pass, I use that a lot, you know, to get to know the city, and stuff like that. (Housing Association tenant)

Well, the fact that they are looking out for my own wellbeing kind of helps me get through. I mean, money’s stressful, especially when it’s tight. So, when you know your landlord is not just, you know, wanting the money through the door every month, he’s actually hoping that you’re okay and you’re able to afford it, it’s reassuring. It helps, you know, keep the stress levels down. (Letting Agency tenant)

**Figure 5.**
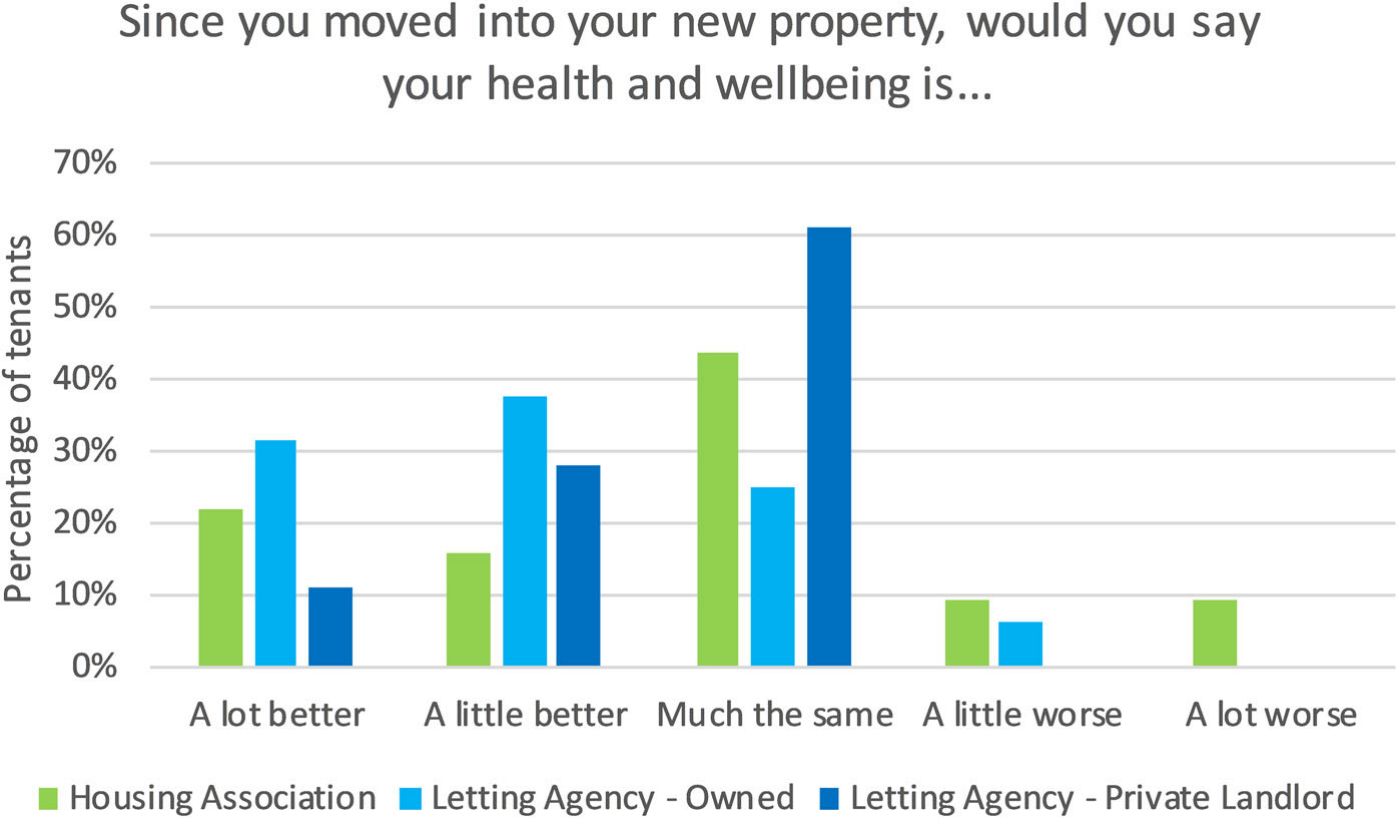
Change in tenants' self-rated health and wellbeing.

These impacts on health and wellbeing were measurable, as illustrated in Figure 5. Notably, the pattern of improvement in health and wellbeing is stronger amongst tenants in Housing Association and Letting Agency-owned properties, by comparison with private landlord properties managed by the Letting Agency. Whist this may reflect a different service experience, as the latter tenants generally do not receive additional tenancy support, it may also relate to better previous housing experiences for the less disadvantaged private landlord tenants.

Indeed, other data suggests that positive housing experiences for disadvantaged tenants can play a role in addressing health inequalities. The World Health Organisation 5-point wellbeing scale (Topp *et al*., 2015), which was used to measure wellbeing at each wave, shows higher mean scores for private landlord tenants at each wave, but a narrowing of the gap by comparison to tenants in Housing Association and, in particular, Letting Agency-owned properties.

This evidence suggests, therefore, that third sector principles shape frontline interactions between tenants and staff in ways that have significant positive impacts on tenants. For the Housing Association, there is some evidence that scale and bureaucracy, perhaps influenced by a pervasion of public sector principles, may undermine these beneficial outcomes in some instances, although state influence in terms of tenancy regulation is experienced more positively. For the Letting Agency, the diffusion of third sector principles through the tenancy support service and approach to tenancy security, creates an experience for tenants in Agency-owned properties on a par with social housing. Whilst private sector principles are clearly more dominant for tenants in private landlord properties, there is no evidence that this undermines tenant satisfaction or wellbeing.

The following section draws the findings together and explores their implications for the examination of hybridity in housing organisations, and for the future of the housing sectors in Scotland.

## Conclusion and implications

The relationships between aspects of hybridity and tenant outcomes are inevitably somewhat complex, given the wide range of factors at play. Changes unrelated to hybridity can occur within organisations, with implications for tenants, whilst the kind of self-rated tenant data used in this study is subject to external influences, not least tenants’ previous housing experiences. Nevertheless, it is possible to identify areas where organisational or modal aspects of hybridity seem to be relevant in shaping impacts on tenants.

For the Housing Association, values established by the tenant-led origins of the organisation place its roots firmly in the third sector (Billis, 2010). Principles emerging from these roots are clearly evident in operational priorities which influence allocation policies, rent levels and customer service standards, which in turn shape the types of tenants who can access tenancies, affordability for tenants and service satisfaction levels. In some instances, these are reinforced by state regulation, such as the role of section 5 referrals in adding homeless households to the tenant population. Moreover, some aspects of particular importance for tenants, such as security of tenure, are heavily shaped by regulation, albeit that they chime with the organisation's core principles and values.

Often, however, the third sector principles are in tension with private sector principles arising from changes to market-based financing, which drive towards higher rents, strict arrears protocols and limited investment in property refurbishment for new tenants. Aspects of bureaucratic structure and processes also seem to play a role in shaping service standards, but it is less clear whether these are driven by market pressures, public sector values arriving with transferred staff, or simply an inevitable consequence of increasing scale. Indeed, all of these factors emerged during focus group discussions with staff at the end of the project, suggesting that there are multiple drivers operating at different points within the organisation. Arguably the protocol-based, somewhat impersonal services experienced by a minority of tenants are evidence of emerging New Public Management principles (Sprigings, 2002; Walker, 1998). However, whether such principles are driven primarily by coercive isomorphism, with state regulation through Scottish Housing Regulator oversight pushing organisations towards similarity, or by a more generalised mimetic or normative isomorphic shift towards private sector styles of management, where Housing Associations are copying best practice or converging as a result of shared managerial culture (Manville *et al*., 2016) cannot be determined from this study.

For the Letting Agency, evidence from the differences between the tenants in owned and private landlord properties, in terms of tenant characteristics and levels of service and property satisfaction, indicate the forms of hybridity within the organisation. Third sector principles encapsulated in the Agency’s social mission clearly drive prioritisation within allocation processes, rent levels, investment in property quality and the central focus on tenancy support. For tenants in properties owned by the organisation, there is a degree of tension between this social mission and market pressures which, for example, preclude the possibility of keeping rents below benefit thresholds. However, these tensions with private sector principles are more obvious in relation to private landlord properties, where the Agency has to balance tenant needs with profitability for landlords, and the wider organisational requirement to maintain this aspect of the business to cross-subsidise tenancy support.

By contrast with the Housing Association, these elements of hybridity are more consciously enacted (Billis, 2010) within the establishment of the Letting Agency. Moreover, these elements were strongly reflected in the final staff focus group, highlighting the extent to which recruitment strategy and management have underpinned organisational values and approaches. Some of the private sector principles operating within the organisation are explicitly designed to support the social mission. By retaining a rent limit above benefit levels for owned properties and allowing private landlord rents to be set at market levels, the Agency compromises on affordability in order to finance investment in property quality and tenancy support. However, it is important to note that this compromise is only partly successful at the current scale, inasmuch as the tenancy support service is only partly funded in this way, requiring additional grant funding.

Perhaps most notably in terms of tenant experiences and outcomes, the Letting Agency's approach to tenure within its own properties demonstrates an implicit form of modal hybridity. Whilst participants in this study were legally no more secure in their tenancy than any other PRS tenant, the level of reassurance given by the organisation made them feel as if they were in a lifetime tenancy, equivalent to social housing.

This study aimed primarily to examine the impacts of hybridity on tenants within each organisation, rather than to compare them. Differences in demographics and previous housing experience make comparisons between the groups of tenants challenging, whilst the complex patterns of hybridity make for particularly convoluted causal connections. Nevertheless, the evidence of relatively greater improvements in health and wellbeing, as well as satisfaction with service and property quality, amongst tenants of Letting Agency-owned properties by comparison with both Housing Association and private landlord tenants suggests some interesting possibilities in terms of modal hybridity. Indeed, the blurring of tenure and rent boundaries from a tenant perspective suggests that hybrid housing organisations within the PRS may have the potential to play an important role in responding to the excess demands on social housing (Powell *et al*., 2015) and the consequent shift of vulnerable households into the private sector (Bailey, 2018). Such blurring of sectoral boundaries generates a range of questions for policy-makers, particularly in contexts such as Scotland where policy appears to be deliberately drawing the PRS closer to social housing. Clearly this also raises questions for the categorisation of housing organisations and products within research.

The evidence from this study also demonstrates that aspects of hybridity can have significant effects on tenants’ housing experience where market or state pressures constrain the social mission of third sector organisations. Where such processes of hybridisation feed through into higher rents, or depersonalisation of services, this can affect not just tenants' satisfaction with their tenancy, but ultimately their wider wellbeing and quality of life. Such findings suggest that research on hybridity in housing organisations needs to extend beyond a focus on structure and strategy (Gruis, 2008; Morrison, 2016; Mullins & Jones, 2015) to understand the implications of organisational changes. Moreover, this study suggests that examining tenant outcomes can help to elucidate how different aspects of hybridity play out within organisations, particularly the ways that pressures at an organisational level may influence the behaviour of frontline staff and therefore the street-level implementation of strategic direction (Lipsky, 1997; Tomlins, 1997). In this respect, there may be considerable value in placing tenant outcomes alongside descriptor, motivator and behavioural variables (Crossan & Til, 2009) to examine and assess hybridity. Hence, hybridity is not merely important for tenant outcomes, but it is also true that tenant outcomes are important for the understanding of hybridity.

### Limitations and further research

This study explores just two specific examples of housing organisations within one national context. Hence, further studies of a wider range of organisations across different contexts would be beneficial to expand the understanding of tenant outcomes of hybridity.

The prioritisation of tenant data within the study also places some limitations on the level of detail in the organisational data. Additional research would be valuable, placing a greater focus on the links between external and internal drivers of hybridisation, the specific patterns of hybridity created, and the ultimate impacts on tenants. Within this, longitudinal explorations of the shifting nature of organisational values and the ‘elective’ elements of hybridity in management decision-making would be useful. Moreover, exploring the impact of regulation and emerging hybridity which may be shifting the social-private boundary in rented housing would be particularly useful for a range of audiences.

## Data Availability

The data underlying this study has not yet been archived in a repository. Parties interested in accessing the data should contact the corresponding author.

## Notes

1 Right to Buy, introduced by the Thatcher government in 1980, gave all Council tenants the right to purchase their home, at a price significantly below market levels.

2 Stock transfer was supported financially by the New Labour governments from 1997 as part of a programme to improve the standard of social housing. Council properties were transferred to Housing Associations where approved by tenant ballots.

3 Note that only some of these changes to PRS regulation had been implemented prior to the fieldwork period for this study – landlord and letting agency registration and regulation had been introduced, whereas the Private Residential Tenancy came in after the research period, although the underlying legislation had been passed.

4 Because of the difficulties with tenant recruitment for the study, a much smaller sample is available from the third organisation, with limited value in relation to issues of hybridity.

5 The attrition in participation rates reflect a range of factors, such as the additional demands of later waves (face-to-face interviews, vs. short telephone interviews at wave 1), changes in tenants’ circumstances, stressful live events, and so forth.

6 MMR provides ‘affordable’ rental property to be rented below market rates, but at rates higher than social housing, delivered by Housing Associations and supported by government subsidy.

7 Local Housing Allowance is the maximum rate set which can be paid in Housing Benefit for PRS tenancies.

8 Individual data (e.g. age, disability) relates to the main tenant who participated in the research interviews. It is important to note that this data relates only to research participants, but that data provided by the organisations suggests that the percentages are broadly reflective of their tenants as a whole, with the exception that Housing Association participants were somewhat younger than Housing Association tenants as a whole.

9 Housing Associations have a duty under Section 5 of the Housing (Scotland) Act 2001 to house statutory homeless people who are referred to them by the local authority unless there is a good reason not to do so.
